# Supplementary material for: A genome-wide scan for candidate lethal variants in Thoroughbred horses
Source: Sci Rep. 2020 Aug 4;10:13153. doi: 10.1038/s41598-020-68946-8 (PMC7403398; doi:10.1038/s41598-020-68946-8)
Supplement: Supplementary file 1 — Supplementary information [file 41598_2020_68946_MOESM1_ESM.pdf]

A genome-wide scan for candidate lethal variants in Thoroughbred horses.

Supplementary Information

Evelyn T. Todd[1*], Peter C. Thomson[1], Natasha A. Hamilton[2], Rachel A. Ang[1], Gabriella Lindgren[3,4], Åsa Viklund[3], Susanne Eriksson[3], Sofia Mikko[3], Eric Strand[5] and Brandon D. Velie[1].

[1] School of Life and Environmental Sciences, The University of Sydney, NSW 2006, Australia.

[2] Racing Australia Equine Genetics Research Centre, Racing Australia, NSW 2000, Australia.

[3] Department of Animal Breeding and Genetics, Swedish University of Agricultural Sciences, Uppsala, Sweden.

[4] Livestock Genetics, Department of Biosystems, KU Leuven, Leuven, Belgium.

[5] Department of Companion Animal Clinical Sciences, Faculty of Veterinary Medicine, Norwegian University of Life Sciences, Oslo, Norway.

*Correspondence and requests for materials should be addressed to Evelyn T. Todd (email: evelyn.todd@sydney.edu.au)

Table S1: Variant frequencies of SNPs 6:38278097 and 6:38278874 for mixed domestic horse from Petersen et al., 2013(1). The total number of individuals in this dataset is 582.

Breed	Sample Size	6.38278097			6.38278874		
		GG	AG	AA	CC	AC	AA
**Akhal Teke**	19	0	0	19	0	0	19
**Andalusian**	18	0	2	16	0	2	16
**Arabian**	19	0	1	18	0	1	18
**Belgian**	19	0	4	15	0	4	15
**Caspian**	16	0	0	16	0	0	16
**Clyde**	24	0	0	24	0	0	24
**Exmoor**	19	0	0	19	0	0	19
**Fell**	19	0	0	19	0	0	19
**Finn**	18	0	0	18	0	0	18
**Fjord**	19	0	0	19	0	0	19
**Frenches-Montagnes**	19	0	2	17	0	2	17
**French Trotter**	17	0	8	9	0	8	9
**Hanovarian**	15	0	1	14	0	1	14
**Icelandic**	19	0	3	16	0	3	16
**Mini**	18	0	2	16	0	2	16
**Mongolian**	19	0	3	16	0	3	16
**Morgan**	19	0	5	14	0	5	14
**New Forest Pony**	14	0	7	7	0	7	7
**Paint**	15	0	1	14	0	1	14
**Puerto Rican Paso Fino**	19	0	8	11	0	8	11
**Mangalarga Paulista**	19	0	5	14	0	5	14
**Percheron**	19	0	0	19	0	0	19
**Peruvian Paso**	19	0	2	17	0	2	17
**Quarter Horse**	19	0	12	7	0	12	7
**Saddlebred**	19	0	2	17	0	2	17
**Shetland**	19	0	2	17	0	2	17
**Shire**	19	0	2	17	0	2	17
**Standardbred**	19	0	3	16	0	3	16
**North Swedish Horse**	18	0	0	18	0	0	18
**Swiss Warmblood**	14	0	1	13	0	1	13
**Thoroughbred**	19	0	5	14	0	5	14
**Tuva**	15	0	4	11	0	4	11

Table S2: Variant frequencies of SNPs 6:38278097 and 6:38278874 in different populations of Quarter Horses (n=137). Data from Petersen et al. (2014)(2)

Breed	6:38278097				6:38278874			
	SNP frequencies			Proportion of heterozygotes	SNP frequencies			Proportion of heterozygotes
	GG	AG	AA		CC	AC	AA	
**Reining (*n*=23)**	0	19	4	0.826087	0	19	4	0.826087
**Pleasure (*n*=23)**	0	16	7	0.695652	0	16	7	0.695652
**Halter (*n*=23)**	0	11	12	0.478261	0	11	12	0.478261
**Working Cow (*n*=24)**	0	21	3	0.875	0	21	3	0.875
**Racing (*n*=23)**	0	13	10	0.565217	0	13	10	0.565217
**Cutting (*n*=21)**	0	17	4	0.809524	0	17	4	0.809524
**Total (*n*=137)**	0	97	40	0.708029	0	97	40	0.708029

Table S3: Allele frequencies of shortlisted variants in high linkage equilibrium with the two SNPs of interest (of 6:38278097 and 6:38278874). Variants were identified from whole-genome sequence data for 90 domestic horses of mixed breeds.

Variant	Minor Allele (B)	Major Allele (A)	B/B	A/B	A/A	r^2	*D′*	Distance from 6:38278097-6:38278874	Annotation
6:38223012	A	AT	2	11	23	0.83	1	55085	Intronic Variant LY49F
6:38264682	T	C	1	16	51	0.81	1	13415	Intronic Variant LY49F
6:38265144	C	T	1	13	46	0.85	0.92	12953	Intronic Variant LY49F
6:38273491	G	A	0	18	50	0.87	1	4606	Intronic Variant LY49F
6:38273498	T	C	0	18	49	0.87	1	4599	Intronic Variant LY49F
6:38273500	T	C	0	18	50	0.87	1	4597	Intronic Variant LY49F
6:38273759	T	C	0	15	52	0.93	1	4338	Intronic Variant LY49F
6:38274367	A	C	0	16	56	1	1	3730	Intronic Variant LY49F
6:38274942	C	T	0	14	44	1	1	3155	Intronic Variant LY49F
6:38276456	C	T	0	15	46	1	1	1641	Intronic Variant LY49F
6:38276742	T	A	0	11	53	0.83	1	1355	3'UTR variant LY49B
6:38276955	A	G	0	15	54	1	1	1142	3'UTR variant LY49B
6:38278097	G	A	0	16	56	1	1	0	Intronic Variant LY49B
6:38278874	C	A	0	15	54	0.93	1	0	Intronic Variant LY49B
6:38281733	A	AG	0	16	51	1	1	2859	Intronic Variant LY49B
6:38282610	A	G	0	16	47	1	1	3736	Missense variant LY49B
6:38284541	C	T	0	16	51	0.93	1	5667	Synonymous variant LY49B
6:38285848	A	G	0	14	53	0.85	0.92	6974	Intronic Variant LY49B
6:38290614	T	A	1	13	49	0.85	0.92	11740	Intergenic variant
6:38292923	T	A	1	14	55	0.86	0.93	14049	Intergenic variant
6:38294589	T	C	1	13	51	0.85	0.92	15715	Intergenic variant
6:38296564	T	C	1	13	53	0.85	0.92	17690	Intergenic variant

6:38297140	A	G	1	14	42	0.86	0.93	18266	Intronic Variant MAGOHB
6:38297149	A	C	1	14	42	0.86	0.93	18275	Intronic Variant MAGOHB
6:38304295	T	A	1	11	43	0.83	0.91	25421	Intronic Variant MAGOHB
6:38319283	*	A	0	12	27	0.81	0.90	40409	Intergenic variant
6:38334772	T	A	0	12	47	0.82	0.91	55898	Intergenic variant
6:38341477	A	G	0	16	48	0.86	0.93	62603	Intergenic variant
6:38341607	A	G	0	15	58	0.86	0.92	62733	Intergenic variant
6:38345686	A	G	0	16	58	0.86	0.93	66812	Intergenic variant
6:38349116	C	G	0	9	12	0.86	1	70242	Intronic Variant LY49C
6:38349743	A	T	1	13	48	0.85	1	70869	Intronic Variant LY49C
6:38351619	C	G	1	15	57	0.80	0.93	72745	Intronic Variant LY49C
6:38352677	G	A	1	9	20	0.89	1	73803	Intronic Variant LY49C
6:38353265	A	G	1	13	51	0.85	1	74391	Intronic Variant LY49C
6:38354110	A	AGTTT AGTTT C	0	16	59	0.86	0.93	75236	Intronic Variant LY49C
6:38358475	AATA T	AAT	1	11	38	0.83	1	79601	Intergenic variant
6:38360920	A	T	1	15	47	0.87	1	82046	Intergenic variant
6:38399815	A	C	0	16	40	0.86	0.93	120941	Intergenic variant
6:38441535	G	C	1	15	52	0.80	0.93	162661	Intronic Variant LY49E

Table S4: Intensity values from microarray data of chorion and chorionic girdle equine embyronic tissue samples. Data from Read et al. (2018)(3).

Tissue	Day of development	Intensity value
Chorionic Girdle	27	6.602977
Chorionic Girdle	27	6.589985
Chorionic Girdle	27	6.62416
Chorionic Girdle	27	6.546489
Chorionic Girdle	30	6.611029
Chorionic Girdle	30	6.639321
Chorionic Girdle	30	6.656763
Chorionic Girdle	30	6.633977
Chorionic Girdle	30	6.561605
Chorionic Girdle	31	6.619057
Chorionic Girdle	31	6.637935
Chorionic Girdle	31	6.651646
Chorionic Girdle	31	6.607523
Chorionic Girdle	31	6.585455
Chorionic Girdle	34	6.568278
Chorionic Girdle	34	6.657548
Chorionic Girdle	34	6.624379
Chorionic Girdle	34	6.656019
Chorionic Girdle	34	6.659633
Chorion	27	6.620722
Chorion	27	6.558723
Chorion	27	6.672482
Chorion	27	6.570658
Chorion	30	6.57291
Chorion	30	6.602505
Chorion	30	6.55263
Chorion	30	6.58852
Chorion	30	6.638503
Chorion	31	6.582505
Chorion	31	6.587206

Chorion	31	6.639011
Chorion	31	6.616271
Chorion	31	6.539944
Chorion	34	6.630667
Chorion	34	6.642043
Chorion	34	6.569519
Chorion	34	6.624507
Chorion	34	6.588266

Table S5: Publicly available whole genome sequence data for domestic horses used in variant calling analysis. The study accession and sample accession relate to the fields under which the sample is listed on The European Nucleotide Archive.

Sample	Breed	Study accession
ERR1527947	Holsteiner	PRJEB14779
ERR1527948	Akhal-Teke	PRJEB14779
ERR1527949	Akhal-Teke	PRJEB14779
ERR1527950	Akhal-Teke	PRJEB14779
ERR1527951	Arabian	PRJEB14779
ERR1527952	Franches-Montagnes	PRJEB14779
ERR1527953	Franches-Montagnes	PRJEB14779
ERR1527954	Franches-Montagnes	PRJEB14779
ERR1527955	Franches-Montagnes	PRJEB14779
ERR1527956	Franches-Montagnes	PRJEB14779
ERR1527957	Franches-Montagnes	PRJEB14779
ERR1527958	Franches-Montagnes	PRJEB14779
ERR1527959	Franches-Montagnes	PRJEB14779
ERR1527960	Franches-Montagnes	PRJEB14779
ERR1527961	Franches-Montagnes	PRJEB14779
ERR1527962	Franches-Montagnes	PRJEB14779
ERR1527963	Franches-Montagnes	PRJEB14779
ERR1527964	Franches-Montagnes	PRJEB14779
ERR1527965	Franches-Montagnes	PRJEB14779
ERR1527966	Haflinger	PRJEB14779
ERR1527967	Koninklijk Warmbloed Paard Nederland	PRJEB14779
ERR1527968	Quarter Horse	PRJEB14779
ERR1527969	Quarter Horse	PRJEB14779
ERR1527970	Quarter Horse	PRJEB14779
ERR1527971	Franches-Montagnes	PRJEB14779
ERR1527972	Swiss Warmblood	PRJEB14779
ERR1545178	Badenwürttembergisches Warmblut	PRJEB14779
ERR1545179	Bayrisches Warmblut	PRJEB14779
ERR1545180	Hannoveraner	PRJEB14779
ERR1545181	Holsteiner	PRJEB14779
ERR1545182	Holsteiner	PRJEB14779
ERR1545183	Oldenburger	PRJEB14779
ERR1545184	Oldenburger	PRJEB14779

ERR1545185	Trakehner	PRJEB14779
ERR1545186	Westfale	PRJEB14779
ERR1545187	Westfale	PRJEB14779
ERR1545188	Swiss Warmblood	PRJEB14779
ERR1545189	Swiss Warmblood	PRJEB14779
ERR1545190	Holsteiner	PRJEB14779
ERR1735862	Thoroughbred	PRJEB14779
ERR2179540	Franches-Montagnes	PRJEB14779
ERR2179541	Franches-Montagnes	PRJEB14779
ERR2179542	German Riding Pony	PRJEB14779
ERR2179543	Welsh Pony	PRJEB14779
ERR2179544	Polish Warmblood	PRJEB14779
ERR2179545	German Riding Pony	PRJEB14779
ERR2179546	Morgan Horse	PRJEB14779
ERR2179547	Holsteiner	PRJEB14779
ERR2179548	Oldenburger	PRJEB14779
ERR2179549	Hannoveraner	PRJEB14779
ERR2179550	Welsh Pony	PRJEB14779
ERR2179551	Arabian	PRJEB14779
ERR2179552	Noriker	PRJEB14779
ERR2179553	Haflinger	PRJEB14779
ERR2179554	Haflinger	PRJEB14779
ERR2179555	Haflinger	PRJEB14779
ERR2179556	Swiss Warmblood	PRJEB14779
ERR2203766	Trakehner	PRJEB14779
ERR2731055	American Paint Horse	PRJEB14779
ERR2731056	Shetland Pony	PRJEB14779
ERR2731057	Icelandic	PRJEB14779
ERR2731058	Holsteiner	PRJEB14779
ERR2731059	Holsteiner	PRJEB14779
ERR2731060	Holsteiner	PRJEB14779
ERR2731061	Akhal-Teke	PRJEB14779
ERR3317814	Deutsches Reitpony	PRJEB14779
ERR3628171	Traber	PRJEB14779
ERR3628172	Traber	PRJEB14779
ERR3628173	Traber	PRJEB14779
ERR3628174	Franches-Montagnes	PRJEB14779
SRR505867	Thoroughbred	PRJNA168142
SRR515202	Thoroughbred	PRJNA168142
SRR515203	Thoroughbred	PRJNA168142

SRR515204	Thoroughbred	PRJNA168142
SRR515205	Thoroughbred	PRJNA168142
SRR515206	Thoroughbred	PRJNA168142
SRR515208	Thoroughbred	PRJNA168142
SRR515209	Thoroughbred	PRJNA168142
SRR515211	Thoroughbred	PRJNA168142
SRR515212	Thoroughbred	PRJNA168142
SRR515213	Thoroughbred	PRJNA168142
SRR515214	Thoroughbred	PRJNA168142
SRR515215	Thoroughbred	PRJNA168142
SRR515216	Thoroughbred	PRJNA168142
SRR515217	Thoroughbred	PRJNA168142
SRR515218	Thoroughbred	PRJNA168142
SRR641364	Thoroughbred	PRJNA184688
SRR641365	Thoroughbred	PRJNA184688
SRR641366	Thoroughbred	PRJNA184688
SRR641367	Thoroughbred	PRJNA184688

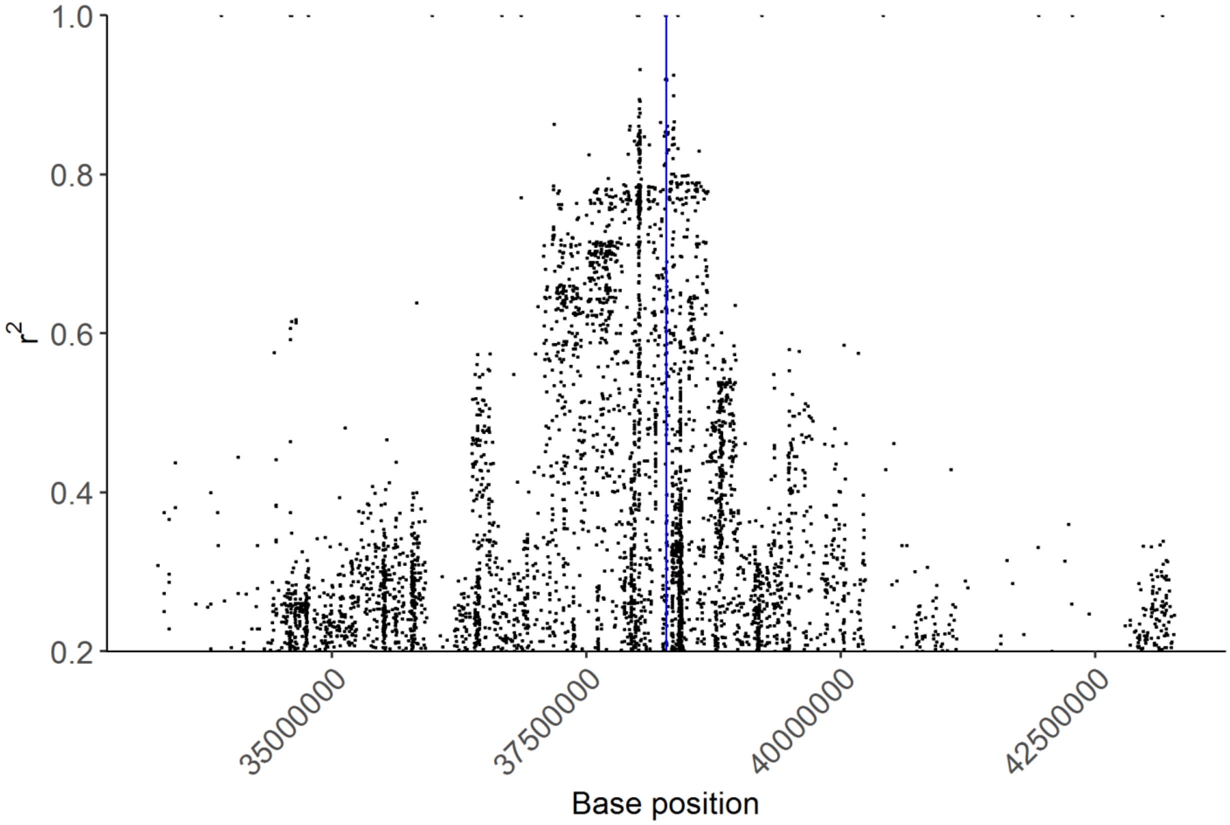

Figure S1: Linkage disequilibrium patterns relative to the markers 6.38278097 and 6.38278874. The positions of 6.38278097 and 6.38278874 are indicated with the blue line. The region spanning 5Mb on either side of the 6.38278097 and 6.38278874 was included in this plot. Variants were called from whole-genome sequence data of 90 horses.

References

1. Petersen JL, Mickelson JR, Cothran EG, Andersson LS, Axelsson J, Bailey E, et al. Genetic diversity in the modern horse illustrated from genome-wide SNP data. PLOS ONE. 2013;8(1):e54997.

2. Petersen JL, Mickelson JR, Cleary KD, McCue ME. The American Quarter Horse: population structure and relationship to the Thoroughbred. J Hered. 2014;105(2):148-62.

3. Read JE, Cabrera-Sharp V, Offord V, Mirczuk SM, Allen SP, Fowkes RC, et al. Dynamic changes in gene expression and signalling during trophoblast development in the horse. Reproduction. 2018;156(4).
